# Notes of a Recent Visit to Several Provincial Asylums for the Insane in France

**Published:** 1851-01-01

**Authors:** John Webster

**Affiliations:** FELLOW OF THE ROYAL COLLEGE OF PHYSICIANS, CONSULTING PHYSICIAN TO ST. GEORGE AND ST. JAMES'S DISPENSARY, ETC.


### Original Communications.

### NOTES OF A RECENT VISIT TO SEVERAL PROVINCIAL ASYLUMS FOR THE INSANE IN FRANCE.

BY JOHN WEBSTER, M.D., F.R.S., FELLOW OF THE ROYAL COLLEGE OF PHYSICIANS, CONSULTING PHYSICIAN TO ST. GEORGE AND ST. JAMES'S DISPENSARY, ETC.

(*Continued from page* 540 *of No. XII. for October*, 1850.)

#### Asylum at Le Mans.

In the sketches given of the four asylums referred to in previous pages, whilst there was much to approve, still some points merited criticism, if not condemnation. The remarks I considered it necessary to make during my narrative, may perhaps seem severe; but as nothing was stated excepting from personal observation, and as I felt more anxious to communicate facts than opinions, even should the statements produce unfavourable impressions respecting some of the institutions described, the fault was not mine, seeing my chief object was to be a faithful reporter. Respecting the asylum which I now propose passing under review, it becomes a very agreeable duty to state, by way of preface, that of all the public establishments for the insane I have recently visited in France, that of Le Mans has certainly given me the most satisfaction. But as this seems like pronouncing a verdict before taking evidence, I will therefore proceed, as in similar cases, to give the data which bear out the above conclusion.

This public asylum is situated close to the banks of the river Huisne, near Le Mans—formerly the capital of the province of Maine, and now the chief town of the department of La Sarthe. Its position is somewhat low, but agreeable, open, airy, and apparently salubrious. Like other newly-established departmental lunatic asylums, it is under the authority of the préfet of the department, as also the minister of the interior, and has a local commission of surveillance, composed of five members, whose orders are administered by a resident Director, and hence dissimilar, in several respects, from some of the establishments to which attention has been hitherto directed. The Le Mans Asylum, in consequence of having been constructed expressly for the reception of lunatics, and under good medical advice, is consequently devoid of many of the defects often inherent to other institutions. The court-yards are spacious, airy, and quite distinct, although of easy communication. The dormitories are cheerful, well lighted, properly ventilated, and of scrupulous cleanliness. The chapel, offices, kitchen, and principal appendages of the establishment, are in the centre; the court-yards for female being on one side, and those for male patients on the other. But without entering into every particular, I have no hesitation in saying, that the whole arrangements of this institution for lunatics, are the best adapted for the objects proposed, of any I have seen elsewhere; and visiting English justices, or architects, before drawing plans for new asylums, might examine the entire structure with profit and advantage, as well in reference to the accommodation for lunatics, as to relieve the pockets of grumbling county ratepayers.

During the year 1849, the following table exhibits the movement of patients at the Le Mans Asylum.

| | | | |
|---|---|---|---|
| Admitted ... ... ... | Males, 41 ... ... | Females, 33 ... ... | Total, 74. |
| Discharged, cured | Males, 15 ... ... | Females, 20 ... ... | Total, 35. |
| Died ... ... ... ... | Males, 8 ... ... | Females, 8 ... ... | Total, 16. |

In reference to the mortality here reported amongst the lunatics, it is instructive to mention that, unlike the other four asylums previously described, not a single fatal case of cholera occurred throughout the whole twelve months; diseases of the chest, on the other hand, being frequently the apparent cause of death amongst the inmates.

On the day I visited this departmental asylum, the total number of lunatics amounted to 258; of whom, 124 were males, and 134 females; the indigent patients being 84 males, and 103 females; thus leaving 40 men and 31 women, or a total of 71 individuals, who paid for their treatment and maintenance, sums varying from 400 to 2,000 francs per annum.

There is only one physician attached to this institution, who, however, constantly resides within its precincts, and also gives his time and exertions exclusively to official duties. This officer, Dr. Etoc-Demazy, is well known for his professional attainments, and as the author of "Researches on Suicide." One *interne* resides likewise in the asylum; so that, should the physician happen to be absent, medical assistance may be always procured in case of any emergency. Besides these two resident medical gentlemen, a surgeon is officially attached to the establishment; whilst the minor details, in reference to patients, are superintended by several benevolent sisters of charity belonging to the order of Evron.

Respecting the all-important subject of restraint, I may state that, on the day of my visit to the asylum, three men and four women had on strait-waistcoats; although some were merely placed under such restraint for a very short period; and no individual was then in the cells, or in any other way confined. One male and two female patients, I also observed, were sitting in arm-chairs to inhale the fresh air in their respective courtyards, who were slightly attached to their seats by a loose band; but neither case must be reckoned as examples of mechanical coercion, seeing the occupants were paralytic and could not move, the belt alluded to being merely to keep them from falling.

Occupations of various descriptions are carried forward with much zeal by the authorities of this institution; indeed, to engage the lunatics in some employment seemed to be the great object pursued. Thus, when going over the gardens and adjoining fields, I saw many inmates busily occupied in gardening, cutting firewood, and preparing foundations for new buildings. At another part, a gang of upwards of a dozen lunatics were finishing a long tunnel they had made under the public highway, in order to connect a field, recently purchased, with the present farm. Other patients, again, were lowering a slope where a washing-house was to be constructed. Throughout the farm—consisting of about fifteen acres, various individuals appeared engaged like ordinary work-people; whilst the industry and orderly conduct then prevailing out of doors gratified me much; as also on noticing, that the labourers always saluted their physician respectfully as soon as he approached, and conversed about their occupations as if they were not insane. Hence, judging only from outward appearances, it seemed as if we were quietly perambulating some agricultural establishment, not the precincts of a madhouse.

Within doors, the same aspect of quietude and occupation prevailed; the female patients being engaged in spinning, sewing, making clothes, washing, knitting, or in the kitchen and laundry. In short, the insane inmates performed much of the household work of the establishment. By these means, the expenses of the institution are not only diminished, the inmates much benefited in their bodily health and mental condition, but they also gain a little money; which adds to their comfort in the meantime, and gives them something besides when they leave the institution.

The labour department is so well organized, and so great is the desire often felt, especially by the male patients, to be sent into the fields and gardens to work, that Dr. Etoc-Demazy mentioned, it was even considered a punishment by many of the poor lunatics, when not allowed to join as usual their fellow-workmen. Judging from the empty appearance of the court-yards and dormitories, I had previously visited, this information was fully confirmed, seeing all these localities were nearly without occupants, whilst few patients seemed either idle or unemployed.

Amongst the inmates, paralytic cases were numerous; but few were classed as idiots, or epileptic patients. As in other similar establishments, a number of cases were considered incurable, their disease being of long continuance. Nevertheless, looking at the ratio of cures, compared with the admissions, the benefits effected seem to have been considerable.

As additional accommodation will soon be completed for indigent patients, and as several new cottages, with gardens, are also about being erected, to receive insane persons who pay for their maintenance, the asylum will then have sufficient space to accommodate 310 inmates. Even now, the dormitories are by no means so overcrowded as I have elsewhere seen, from deficient accommodation; and as none of the wards in this asylum contain more than 12 beds, whilst several have only 8; and farther, as there are generally windows on each side of the apartments, the dormitories appear cheerful, better ventilated, and are consequently, I think, more salubrious, than in many other institutions.

Comparisons are often odious, even if just; still, the authorities of this asylum, both medical and lay, deserve great praise for the unwearied and successful efforts they constantly make, to alleviate the severe afflictions of those unfortunate victims of mental alienation committed to their charge. The facts narrated in this brief notice, and the impressions I received during my recent visit, made me form a very high estimate of the "Asile de la Sarthe;" which appeared at the time, as afterwards, one of the best constructed and most judiciously-managed lunatic institutions, I have anywhere had an opportunity of examining; especially in reference to employing the insane inmates. When accompanying the zealous resident physician through the various divisions of this establishment, whose efforts have contributed so materially towards its present excellent condition, I said as much to him personally; and although not of much value, I have again much pleasure in also publicly expressing the same sincere opinion, through the medium of Dr. Winslow's *Psychological Journal.*

### Asylum at Blois.

Similar to the institution at St. Gemmes, this asylum is not yet completed; many of the court yards and appurtenances being still in progress. The building was commenced in 1841; and when all the dormitories are erected, will afford accommodation for 350 lunatic patients. The situation is well chosen, being open and airy besides possessing an extensive prospect of the neighbouring country, especially over the southern bank of the Loire; and although in the vicinity of Blois, the establishment is nearly as secluded from observation as if many miles distant. Similar to many other French lunatic asylums, the supply of water is at present defective; but this want of so essential an element will be effectually remedied, should the contemplated improvements for supplying the city of Blois with that most necessary article, for domestic and other purposes, be actually carried forward by the municipality.

The general plan of this institution bears some resemblance to the asylum at Le Mans; the court-yards are judiciously arranged, and decidedly more cheerful than many earlier constructed erections. Already, parterres of flowers have been formed in the enclosures; and when all the dormitories, with other conveniences, are finished, I feel assured this public asylum, intended for the reception of lunatics especially belonging to the department of the Loir and Cher, will merit inspection, and obtain approval.

During the year 1849, the following statement shows the movement of patients; but for the reasons just stated, and its limited extent, the table does not possess the same importance as the reports received from other institutions.

| | | | |
|---|---|---|---|
| Admitted ... ... ... | Males, 26 ... ... | Females, 60 ... ... | Total 86. |
| Discharged, cured . | Males, 13 ... ... | Females, 10 ... ... | Total 23. |
| Died ... ... ... ... | Males, 8 ... ... | Females, 7 ... ... | Total 15. |

Amongst the deaths, several arose from cholera, and chiefly during August; but none after the 27th of that month. Again, in reference to the present population, its amount may be reported as almost now in a state of transition; seeing sixty new patients are expected to arrive very shortly, the dormitories being already prepared for their reception. However, on the day of my visit, the actual number in the asylum was 124 inmates; consisting of 46 male, and 78 female lunatics. Amongst the other peculiarities noticed at this institution, and similar to those mentioned when describing St. Gemmes, the resident physician also combines the attributes of director. This arrangement has certainly advantages in an asylum like the present, where many new works are in progress; as the eye of a medical practitioner, conversant with insanity, is better able to direct the proposed improvements, and necessary alterations in the building, than any other individual. Nevertheless, there are many serious objections to the junction of two such important and distinct offices as resident-physician and director in a lunatic asylum, especially where the patients are numerous. However, on this subject I shall speak more fully, in a subsequent paragraph.

Dr. Billod—an accomplished physician, and well known for his medical works—now occupies the responsible offices of director and resident medical officer of the Blois asylum. That he performs the various duties of these appointments well and satisfactorily, cannot be better illustrated, than by the statements I shall have the gratification of soon making, as the result of my own inquiries and observation at this institution. Besides the physician, one interne is also attached to this institution; but at present that officer is absent, for the purpose of accompanying to the asylum a party of the lunatic patients, whose arrival, as already mentioned, is daily expected. The interne's duties are, however, performed in the interval by an insane patient resident in the asylum, who was formerly a medical practitioner. This unfortunate individual accompanied Dr. Billod and myself, the morning I went round the wards with that gentleman; and it is interesting to state, that he wrote down all the orders of the physician, and otherwise conducted himself as if he had been the real "locum tenens."

Having referred to the acting deputy-interne being a lunatic, I ought also to mention, as illustrations of the benefit, and even safety, of sometimes employing insane persons in various duties; and farther, of giving them official occupations—of course, where such proceedings are considered safe and judicious,—that the present secretary of the asylum is a lunatic; a patient also superintends the horse employed in driving the machine, for pumping water to the various reservoirs; another insane patient takes care of the same horse in the stable; the dairy woman, now in charge of the cows, is insane, and likewise the gardener; a carpenter, who acts as foreman, a shoemaker, employed to teach others, and the person who is at present under-bailiff, having several other lunatic labourers to superintend, are all "non compotes mentis." Further, and which ought not to be forgotten, one of the male attendants, who accompanied Dr. Billod, myself, and the insane interne already mentioned, through the epileptic ward, was likewise a lunatic. These important facts are highly interesting; and I have been thus particular in now recording all the circumstances, as they admirably illustrate the system constantly pursued at this institution; namely, of giving work and employment to every lunatic inmate, wherever practicable.

A recent regulation in the management of the Blois Asylum was subsequently communicated, which I must specially record, as it is both useful and deserving of imitation; namely, the authorities now receive no new attendants into the establishment, unless they understand some trade or handicraft; so that every subordinate official and servant may be qualified to teach patients the requisite occupations. This excellent system not only proves beneficial to the institution in pecuniary respects, but it encourages labour amongst the lunatics, which is highly advantageous, and improves their bodily health, as well as mental condition; whilst they thereby may also gain some money for present comforts and future contingencies.

As might be expected, various occupations are carried forward in this asylum, especially out-of-door employments. In the adjoining farm—which contains at present about fifteen acres, but will soon be augmented to twenty-five by a new purchase—agricultural and horticultural employments are pursued most zealously. When I walked over the enclosure, several labourers were engaged in excavating the foundations of a new court-yard for the agitated patients, and others were carrying the earth from different sites in wheelbarrows, wherewith to form a large mount in the centre of one of the fields, in order that the residents might have a fine and extensive panoramic view of the neighbouring country. Various inmates were also trimming walks in the garden, and so forth. In fact, every person out of doors seemed busy; and the general aspect of the place looked very unlike a lunatic establishment. Again, within doors two weavers were busy at their loom; also, carpenters and a shoemaker. Many female patients—some of whom were also engaged in the fields, as is often seen in-

France—were busily occupied in sewing, in knitting, in the kitchen, or in household work; and others in preparing clothing for the inmates, which essential articles were all made upon the premises. Bodily occupation for the mind diseased being here considered a great adjuvant in the treatment of lunatics, it was carried out to the utmost limit; and Dr. Billod stated, that at least four-fifths of the whole residents were usually employed.

In corroboration of the beneficial effects produced upon the insane by bodily labour, this gentleman also informed me that he had frequently remarked the patients did not sleep so soundly, and the dormitories were often more noisy during Sunday nights than any other period of the week; and this effect he ascribed to the circumstance, that on Sundays the various occupations being interrupted, the whole day was passed by the inmates in comparative idleness. Dr. Billod has made this observation so frequently, from visiting the dormitories on purpose during different nights, that he had no doubt upon the subject; and now communicated the fact as the result of repeated observation and experience, which well deserves the special notice of other psychologists.

As might be anticipated from the details previously reported, mechanical restraint is very little employed at this institution, compared with other places in France; and on the day of my visit not a single male patient was in any way confined. Three female lunatics were, however, in strait waistcoats, one being also tied to her bed, and in a great state of excitement; another of the above had her legs also tied together; but the third female patient was merely in demi-camisole, her hands being quite free. No other lunatic was restrained in any way, and the cells were all empty. I saw no iron bars in the windows; and altogether the general appearance of the patients in this asylum was satisfactory; the women, with the above exceptions, being even quiet and orderly.

Many of the inmates were incurable; some being idiots, a few cretins, and a considerable number of epileptics, with homicidal tendencies. Besides the above formidable varieties of mental disease, three patients were affected with general paralysis, one of whom was a female. Recently, M. Billod had a paralytic patient, who, amongst his other ideas of grandeur—so peculiar to this complaint—at one time considered himself to be a general in chief commanding the army in Italy; then he became Proudhon; afterwards Blanqui; subsequently, Pierre Leroux; Louis Blanc; President of all the Clubs; Secretary of Ledru-Rollin; and latterly, when the physician refused to open the doors of the asylum to allow him to depart, he appointed himself director, having first, in imagination, dismissed that gentleman from both the offices he holds in the institution. Seeing this form of mental, as well as physical disease has become of late more common than formerly, and often baffles every kind of treatment employed, it therefore seems peculiarly interesting to mention, that one of the male attendants at present on duty had been affected with this serious malady, and seems now apparently cured, although the symptoms, at one time, were of an unfavourable character, as shown by the following outline of the case, given in the words of M. Billod. The patient was 33 years of age, of a nervo-sanguineous temperament, and has a mild, open, and intelligent-looking countenance. The symptoms at first were those of acute mania in the most decided form, with extreme agitation and general delirium; the predominant ideas having reference to politics and ambition. He believed the other patients in the asylum were all members of a political club, to whom he often addressed speeches. He desired to regenerate society, and to make every person rich, whilst he believed his own fortune incalculable. There was great excitation of his intelligence, of sensibility, and of will; with such constant wakefulness, that he scarcely ever slept. His speech was embarrassed, tongue tremulous, the muscles of his face being often affected by spasmodic contractions; whilst his gait was vacillating, although generally in movement. The disease continued unabated till the night between the 18th and 19th of last August, when this patient was attacked by cholera—then prevalent in the asylum—whereupon the delirium seemed alleviated. Ultimately, the other symptoms became less pronounced, and in a few days he was able again to resume labour in the fields, which proved highly beneficial; and recently he has been appointed one of the infirmary attendants.

At the present moment, there are not many private or paying patients in this asylum, the accommodation being very limited for such inmates. As it is, however, proposed to erect eight additional apartments, with a private garden, for male patients of the better class; and a similar number on the female side, an increase may be expected; when the payments will be from 600 to 1200 francs annually.

Amongst the insane, physicians occasionally find considerable difficulty in inducing particular patients to take food, when artificial methods become absolutely necessary. The sound, in many of these cases, is employed for that purpose; although other mechanical means have likewise been recommended, some of which are very ingenious, and are often effectual. Dr. Billod having paid great attention to these distressing cases, has recently invented a species of gag made of metal, which he introduces into the mouth of the lunatic. By compressing the nostrils, at the same time that food is conveyed with a spoon through the machine, the patient so treated must swallow whatever comes in contact with the pharynx, whilst a valve empties the spoon, and prevents regurgitation. Dr. Billod spoke highly of the utility of his instrument; and as it will soon be made known to the profession, practitioners can then judge of its efficacy.

When alluding to the employment of insane patients in out-of-door labour, mention was made of a mound, then in course of construction, for the purpose of affording the lunatic inmates an extensive prospect of the adjacent fine country. Wherever the grounds of an asylum permit, and the view is not restricted by near objects, such an elevation should be always made; as it has been found both agreeable and beneficial. A distant view of a beautiful country, presenting a varied prospect, has a tranquillizing effect upon the mind of many lunatics; besides being an excellent mode, as also an inducement for patients to take exercise. It has, however, been observed, by practical physicians, when the mounds are easily overlooked by neighbouring dwellings, or if strangers outside can be distinctly recognised by the lunatics within, then injurious consequences may supervene, especially, if erotic passions characterize the patient's mental malady.

Before taking leave of the asylum at Blois, and of its able physician-director, who is so zealous and successful an advocate for employing the insane wherever possible, or compatible with their safety and improvement, it ought to be distinctly mentioned, that no accident whatever has happened to a single patient at this asylum from any occupation, although carried forward most perseveringly. This fact is conclusive; and, conjoined with others of a similar kind, elsewhere reported, all scepticism on such points must now give way before knowledge and experience.

ORLEANS ASYLUM.

This institution being situated in the city of Orleans—the capital of the department of the Loiret, is so surrounded by houses on almost every side, that, from the upper windows of several, the court-yards of the patients may be easily seen, and conversations even held with the inmates. Besides this defect, not having been originally constructed as an asylum for lunatics, and some of the recent additional buildings being injudiciously made within a very limited space, and which it appears impossible to enlarge or improve, without purchasing many adjoining houses and streets, the faults inherent to this institution can never be corrected effectually. The only judicious remedy would be, to remove the establishment altogether to a more open and airy situation. Such a step seems even more necessary, seeing it is now conjoined with several other charitable institutions, having thereby a very large aggregate population.

Within the same precincts, and under a common board of administration, the present general hospital of Orleans comprises—1st, a workhouse, having, the day I visited this establishment, 310 paupers: 2nd, the Hôtel Dieu—or civil hospital, with 300 patients: 3rd, a refuge for orphans, of whom 80 were then in town, besides 819 in the country: 4th, a receptacle for diseased "filles publiques;" and, 6th, the lunatic asylum, which then contained 521 patients. The different inmates of all these establishments, including resident officials, and domestics, augmented the present population, I was informed, to 1550 individuals, which require an expenditure of at least 550,000 francs per annum.

Notwithstanding the large number of insane patients constantly resident in the lunatic division of this establishment, the medical staff is now more limited than in most other insane institutions of equal magnitude; seeing one resident physician takes charge of all the patients, whom he visits daily, without the assistance of any interne; although there ought to be two, if not three, considering the laborious duties performed. There is doubtless an adjunct medical officer domiciled in Orleans, who visits periodically; still the responsibility rests with the resident physician, Dr. Chambeyron, a practitioner of much experience in mental diseases, and who has filled similar appointments elsewhere. Having upwards of 500 lunatics constantly under surveillance, and many of these being engaged at a farm attached to the asylum, Dr. Chambeyron has full occupation for his time, which must be dedicated wholly to the inmates, as he is prevented from engaging in private practice.

During the year 1849, the movement of insane patients was thus reported:—

| | | | |
|---|---|---|---|
| Admitted ... ... ... | Males, 71 ... ... | Females, 66 ... ... | Total, 137. |
| Discharged, cured... | Males, 20 ... ... | Females, 11 ... ... | Total, 31. |
| Died ... ... ... ... | Males, 48 ... ... | Females, 45 ... ... | Total, 93. |

Comprised in the above 93 deaths, it ought to be stated, that 32 persons died by cholera, 11 being males and 23 females; the greater proportion of which occurred in the month of July, when the recent epidemic prevailed extensively.

Believing a detailed statement of the various diseases assigned as the immediate cause of death in the total 93 patients now reported may be interesting to professional readers, I subjoin a classified statement, containing particulars which will, doubtless, be considered more important, seeing the table illustrates generally, not only the pathognomonic character of all cases ending fatally, but likewise forms an instructive exposition of the physical maladies attacking the inmates of a large French lunatic asylum, in which the mortality proved considerable.

*Classification of the ninety-three deaths recorded in the Lunatic Asylum at Orleans, during* 1849.

| Diseases of Head. | Males | Fem. | Total. |
|---|---|---|---|
| Arachnitis | 1 | — | 1 |
| Congestion | — | 2 | 2 |
| Epilepsy | 6 | 2 | 8 |
| Fracture of skull by a fall | 1 | — | 1 |
| Ramollissement of brain | 9 | 1 | 10 |
| | 17 | 5 | 22 |
| **Diseases of Chest.** | | | |
| Asphyxia by strangulation | — | 1 | 1 |
| Inflammation of lungs | 1 | 3 | 4 |
| Pulmonary apoplexy | 1 | — | 1 |
| Pulmonary catarrh | 2 | — | 2 |
| Pulmonary consumption | 3 | 2 | 5 |
| | 7 | 6 | 13 |
| **Diseases of Abdomen.** | | | |
| Cancer of uterus | — | 1 | 1 |
| Cholera | 11 | 21 | 32 |
| Dysentery | 2 | — | 2 |
| Inflammation of bowels and stomach | 5 | 7 | 12 |
| Inflammation of mesentery and peritoneum | 1 | 1 | 2 |
| | 19 | 30 | 49 |
| **Diseases of uncertain Seat.** | | | |
| Cachexia and decrepitude | 2 | 1 | 3 |
| Erysipelas | 1 | — | 1 |
| Inanition | — | 1 | 1 |
| Small-pox | — | 1 | 1 |
| Typhus fever | 2 | 1 | 3 |
| | 5 | 4 | 9 |
| Total of deaths by all causes | 48 | 45 | 93 |

According to the above statement, diseases of the abdominal viscera proved, in consequence of the recent epidemic, more fatal than any other class of maladies; gastritis and enteritis, after cholera, being in this division the most common cause of death, 12 fatal cases of that description having occurred during the year. Next in point of frequency rank diseases of the head; and in this category 22 deaths are reported, ramollissement of the brain being the most frequent cause, as shown by the fact, that of this formidable and generally incurable alteration of structure there were eight examples. Epilepsy constitutes the second most numerous cause, of which the total fatal cases amounted to eight; and it is instructive to observe, respecting this class of affections, that amongst the whole 18 deaths from both the above diseases, 15 occurred in male patients—viz. nine by ramollissement, and six by epilepsy; whereas only three women died from similar morbid affections. By pectoral complaints only 13 deaths are reported, five being from pulmonary consumption, and four from inflammation of the lungs. The fact of not more than five fatal cases of consumption being met with, amongst the whole 93 deaths reported, forms rather a striking feature, especially, if compared with the mortality often observed from the same disease, in the lunatic establishments of Great Britain, where consumption prevails very frequently. For instance, at Bethlem Hospital, in which lunatic institution 24 deaths occurred during the year 1849, amongst an average population of 405 insane patients, one-sixth of the whole, or four, were produced by phthisis; whereas at the Orleans Asylum, the comparative ratio was only one-eighteenth of the total ninety-three deaths reported. Again, by other diseases, or those designated of uncertain seat, the proportion of deaths at the Orleans Asylum, in 1849, was less than in the three previous divisions, there being only nine fatal cases, of which three were produced by typhus fever, and three, according to the official return I received, by cachexia and decrepitude, but which would be perhaps denominated in England, by the antiquated and indefinite phrases—"old age and decay of nature."

With reference also to another interesting point, namely, the period of life when death supervened in the 93 fatal cases now reported; it is instructive to state, that 14 of the above number occurred in patients under 30 years of age; nearly one-half, or 45, in the prime of life, being then 30 to 50 years old; 18 from 50 to 60; nine from 60 to 70; and four from 70 to 80—besides which, three patients—two men and one woman, were stated to be from 80 to 90 years old at the time of their death; thus showing that some individuals, although insane, may yet attain great longevity.

Respecting the type or general character of the mental maladies affecting the 521 inmates recently under treatment in this asylum, which then consisted, as reported in a former paragraph, of 246 male, and 275 female lunatics, I would next direct attention to the following table, compiled from official documents, as it supplies some rather interesting, if not instructive information on such points; besides being a good index of the ordinary forms of insanity usually met with, in a large provincial establishment for lunatics in France.

TABLE *showing the Type of Mental Disease which affected the* 521 *Insane Patients, under Treatment at the Orleans Asylum.*

| INCURABLE PATIENTS. | Males | Fem. | Total. |
|---|---|---|---|
| Idiocy and imbecility | 44 | 38 | 82 |
| Do. do. complicated with epilepsy | 22 | 14 | 36 |
| Mania complicated with epilepsy | 20 | 20 | 40 |
| Mania of long continuance | 64 | 55 | 119 |
| ——— | 150 | 127 | 277 |
| PROBABLY INCURABLE PATIENTS. | | | |
| Inveterate mania | 70 | 95 | 165 |
| Periodical mania | 2 | 4 | 6 |
| ——— Total | 72 | 99 | 171 |
| CURABLE PATIENTS. | | | |
| Acute dementia | 4 | 5 | 9 |
| Acute monomania | 6 | 16 | 22 |
| Acute mania | 14 | 28 | 42 |
| Total | 24 | 49 | 73 |
| General Total | 246 | 275 | 521 |

From the above data it consequently appears, that only seventy-three patients, or less than one-seventh of the entire number, are classed as curable; whilst in the remaining 448 scarcely any hope of amelioration could be entertained. This fact at once explains the small amount of cures effected last year, as also the numerous deaths reported. Even to have discharged thirty-one individuals convalescent, was creditable to the physician, especially, when it is stated, that only sixty-one curable patients were admitted in 1849; whilst the locality, with other adverse circumstances alluded to previously, are at the same time taken into consideration.

The large proportion of epileptic patients, now resident in the Orleans Asylum, deserves also special notice; the amount being forty-two males and thirty-four females, making a total of seventy-six, who are all considered incurable. The number of idiots and imbecile patients is likewise remarkable; eighty-two cases of this description—consisting of forty-four males and thirty-eight females, being now in the wards. Such a large collection of unfortunate human beings, as the above 158 idiotic and epileptic patients, deserves attention, as the amount shows the great frequency of the above forms of mental diseases, in this part of France. Indeed, according to report, epilepsy is of so frequent occurrence amongst the inhabitants, that several quacks, and even priests, enjoy at present considerable reputation *in curing* this generally incurable disease; which always has, and ever will be, a source of gain to charlatans amongst an ignorant and superstitious population.

This table exhibits, besides, another feature which deserves notice, namely, the large number of incurable male patients compared with female lunatics in the same condition, although the aggregate amount of insane men in the asylum is less than that of the other sex, 150 incurable male lunatics being now on the register, with only 127 females of a similar description.

It likewise deserves especial remark, that the incurable men are not only more numerous than the insane women of the same category, but the number of curable male lunatics under treatment does not reach to half the amount of curable females; seeing twenty-four of the former sex to forty-nine of the other are reported in the above table. These facts, along with the fewer insane males admitted in 1849, prove the inveteracy of insanity amongst men, although the disease is less frequent.

General paralysis, as in some other institutions, seems also of rather frequent occurrence in this portion of the Republic; the number of lunatics now in the asylum, affected with this peculiar form of malady, being sixteen male and three female patients; thus indicating that here, as elsewhere, this disease is always met with in much greater proportion amongst men than women, although the symptoms are most intractable in both sexes, and seldom yield to any remedies, unless at the very commencement of so direful a malady.

When going round the dormitories and various court-yards, the female patients were then very noisy, and appeared quite as much agitated as in any French asylum I have ever visited; the men being also a good deal excited, but certainly less violent than the female lunatics. Further, the proportion of patients under restraint, on the day adverted to, was likewise considerable; as shown by the following enumeration, noted at the time of my perambulations, in order to avoid exaggeration. Eight male patients had strait-waistcoats, of whom two were likewise strapped to their beds; and seventeen female lunatics were in camisoles, two of these being, besides, tied to their beds; whilst two were also shut up by themselves in the court appropriated to agitated female patients.

This division of the asylum stands much in need of improvement, the cells being badly ventilated, unglazed, having stone walls, and are very comfortless. Indeed, the entire locality is altogether unfitted for lunatics, and about the worst of the kind I have ever entered; whilst, from its original defective construction, the faults, as in some other parts of the building, appear irremediable. Nevertheless, it is only just to the authorities to add, that several of the ordinary court-yards are by no means badly constructed, having also pretty parterres, with enclosures of flowers; besides which, a new dormitory,—recently finished, seems superior to the old, at the same time, that others were then undergoing improvements.

As might be expected from the above facts, and other causes, very few private or paying patients are at present under treatment in this asylum. Indeed, the accommodation for such inmates did not appear extensive. Amongst the 521 lunatics now under treatment, only thirty-two inmates, consisting of eleven male and twenty-one females, are classed as voluntary patients, who pay for their maintenance and lodging from 440 to 1200 francs per annum; the remaining 489 residents being all indigent lunatics, or, to employ the distinctive French designation, they were insane patients who had been placed in the asylum, "d'office."

Most of the wards in this institution are not only very confined, but badly ventilated; and were, at my visit, overcrowded with beds, owing to the limited spaces, as also the very large number of lunatics now accumulated together. In consequence of the great deficiency of accommodation, a division of the patients now inhabit some old houses attached to a farm belonging to the hospice, which contains about twenty-five acres, where the inmates are occupied in agricultural and horticultural employments. In addition to the means here afforded, occupations are likewise promoted at the asylum itself, the females being engaged in household work, sewing, knitting, and other employments; the males in the gardens, at handicrafts, and various trades adapted to their situation; but occupying the lunatics did not seem to be carried out either as zealously, or to the same extent, in this asylum as I have noticed in other similar establishments. On this subject one defect may be mentioned, as it chiefly arises from injudicious domestic arrangements; viz. no kitchen being specially appropriated for the lunatic department, all the food required must be brought from the common cuisine; and as the lunatic patients are often sent to carry the provisions, it thus happens, that they mix with the inmates of other divisions of the hospital, which is very objectionable, as I can state from personal observation. Every institution for the insane should be entirely separated from any other charitable establishment, proper classification of the inmates being also of the highest importance; and no court-yard should ever overlook another; still less, should the inmates be seen from neighbouring houses, or be able, on their part, to converse with the occupants of such localities.

Believing enough has already been said respecting the Orleans Lunatic Asylum, I will only now add, that it requires much improvement, if not transference to a separate and more open situation. Such a proceeding would be highly judicious; and as it is the only public provincial establishment for insane patients belonging to the three departments of the Eure and Loir, the Loiret and the Eure, the councils-general of these important districts of France, ought no longer to rest satisfied with the present condition of this institution, which seems far inferior, in point of accommodation, to many others in the country. Should the public authorities, whether local or central, wish to be convinced respecting this important feature, they need not go farther to obtain information, or to observe the beneficial results of experience, than to the neighbouring asylums at Auxerre, or Blois, and especially the institution at Le Mans, whose excellent example they might advantageously imitate.

### Saint Yon Asylum.

This institution derives its name from a M. de Saint Yon, who in 1615 possessed the property. In 1670, M. De Bois-Dauphin became the proprietor, by whom it was converted into a monastery for females and a general hospital, still retaining the original name of Saint Yon.

In 1825, the building was made a receptacle for lunatics, and has continued so till the present time; being specially appropriated to receive insane patients belonging to the department of the lower Seine. This asylum is situated in the Fauxbourg of St. Sever, on the south side of the river, and close to the city of Rouen, the ancient capital of Normandy. The position is good, although low, and rather near the neighbouring houses; still, the foundation being gravel, and having about twenty acres of gardens and fields adjoining, it possesses many advantages not always found in other establishments. The court-yards for female patients are on one side of the domestic offices, and those for the male lunatics on the other; both being quite separate. The pavilion, containing the baths, occupies the centre building; whilst a steam-engine supplies water to this important appendage in all French asylums, as also to the culinary and other departments.

Saint Yon is one of the largest provincial lunatic asylums in France, and was also amongst the first departmental insane institutions established. It is, besides, one of the best conducted in the country; and contained, at the period of my recent visit, 739 insane inmates, 261 being male, and 468 female patients; thus giving a considerable excess of the latter sex over the former, much more so, indeed, than I have noticed in any of the other asylums previously described. Two physicians are attached to the institution, having entire charge of the patients, whom they visit daily. Dr. De Smyttère takes charge of the male, and Dr. Mérielle of the female department; but only one of these physicians now resides in the asylum, namely, Dr. De Smyttère, the other being a practitioner of repute in Rouen. Each of these gentlemen has two internes attached to their respective divisions; two of which, very useful medical officers, in every asylum, must be always on the premises, in case of any emergency; and all the four being resident in the establishment, the official staff seems adequate for every purpose.

Like most provincial public lunatic institutions in France, Saint Yon receives private or paying patients. The number at present is, however, not considerable; and they are lodged in separate quarters, the ladies in a building, with their own garden, on the one side; the gentlemen, who seemed equally well accommodated, occupied another quarter; both being quite distinct from the pauper patients. The pensions vary from 1000 to 1500 francs per annum, according to the accommodation, with 750 francs additional, should the patient desire the services of a special attendant. In reference to this department, it may be worth mentioning, that the building appropriated for private male patients, was constructed by an English architect, and has a separate garden, with a bowling parterre for the amusement of the pensioners.

When perambulating the numerous court-yards and dormitories of this large asylum, the inmates generally seemed much more quiet, compared with those I saw in 1842, during my previous visit to this institution; whilst both the male and female patients now exhibited less excitement than appeared to me at that period. This improvement was especially apparent in reference to the diminished application of the strait-waistcoat, compared with former usage; according to my distinct recollection. The total number of females now mechanically restrained, amongst the whole 468 lunatics of that sex, being seven; whereas of the 261 male patients, only three had camisoles; and I saw no inmate whatever confined in solitary cells, or mechanically restrained in any other manner. This report of the restraint now employed differs from my former observation; and shows the progress made in a question which is so interesting to every Englishman, and also now warmly supported by many French practitioners.

During the year 1819, the movement of insane patients at Saint Yon was as follows:

| | | | |
|---|---|---|---|
| Admitted . ... ... | Males, 100 ... ... | Females, 104 ... ... | Total, 204. |
| Discharged cured . | Males, 39 ... ... | Females, 37 ... ... | Total, 76. |
| Died ... ... ... ... | Males, 56 ... ... | Females, 53 ... ... | Total, 109. |

Amongst the above deaths, nearly one-third, or thirty-three individuals, died by cholera; the sexes being almost equally divided. Besides these casualties, twenty-four male and fourteen female patients were removed from the asylum, previous to their cure being effected.

When visiting the various divisions of this institution, along with the attending physician, I was much pleased with the internal discipline of the entire establishment. Thus, on the female side, those who are able to leave their dormitories, assemble in the middle of the court-yards, and seat themselves on chairs in a circle; so that the physician can thus minutely examine every individual without much trouble, or the chance overlooking any patient, which might be the case, were they dispersed over the enclosure. But in addition to the facility of thus seeing every person, the discipline of making all the female patients seat themselves in regular order, imposes upon them some restraint to remain quiet, at least during the physician's daily visit, which thereby becomes an important aid in their treatment; for if once a lunatic is made to behave properly, the other means employed by the practitioner will likely prove more efficacious. Again, on the men's side, the same principle is followed; but with this difference, that the male patients all stand up in single file, as if at an ordinary military parade; whilst the physician, accompanied by the interne, and other officers, or any visitor who may be present, walk along in front, in the way a general officer with his ataff would inspect a regiment. The same mode of visiting and inspecting the patients is now adopted in many other lunatic asylums; and considering the large numbers frequently congregated together, the plan is judicious; whilst those inmates who are unable to walk, or may be in bed, or in the infirmary, are, of course, afterwards specially examined in their different localities.

The female dormitories seemed exceedingly crowded; and great difficulty was consequently experienced, in accommodating all the patients of this class, whom the authorities have been obliged, from necessity, to admit. Every available corner had therefore an extra bed; and even then, it has been found impossible to admit every applicant. Notwithstanding this overcrowded population of the female wards—generally so noisy in France—the agitation was less than I have noticed elsewhere; whilst the male patients were as usual, more quiet than the female.

This institution seemed well managed in all its domestic, culinary, and vestiary arrangements; whilst the discipline established by the medical officers, appeared excellent. The physicians have almost unlimited or despotic power in this asylum, to regulate everything respecting the medical treatment of the inmates; and they cannot be interfered with by any concurrent, still less, an inferior jurisdiction. The dietary, clothing, occupations, amusements, and recreations of every kind, are all directed by the attending physician; and no attendant whatever is permitted to order anything in reference to treatment or occupation, out of the usual routine, without his express sanction; nor is the friend or relative allowed to visit any lunatic, or the patient to leave a particular division, unless by the specific authority of the medical attendant; besides which the sisters and all servants must obey every mandate emanating from their superiors, especially those issued by the physician.

The patients appeared well fed and clothed, the women in the habiliments they usually wear; but the male patients, excepting those of the upper class, were nearly all clad in an uniform manner. This may suit the French national character, but to my mind, the plan seems objectionable, being too monotonous, besides having more of a military appearance than I should consider desirable.

The same uniformity of dress prevails in other establishments, but it is not so rigidly enforced as at Saint Yon. The system certainly promotes economy; but, amongst persons affected with mental alienation, the custom of clothing every patient alike, whatever may have been the previous tastes and occupation, or present state of mind and feelings of the individual, seems of doubtful utility; and ought not to be adopted in a limited, but still less, in any large asylum containing indigent lunatic patients.

Occupying and amusing the insane patients is considered of essential importance at this institution, where every effort is made to engage the lunatic inmates in some bodily employment. This object was perseveringly and successfully carried forward by M. Parchappe, when chief physician of Saint Yon; and the same very useful system is still pursued by his present able successors. To specify minutely the various trades and handicrafts in which patients are now engaged, seems superfluous; for to do so now, would be but to repeat many remarks similar to those already often made, when speaking of similar establishments. I will therefore at present only say, the asylum at Rouen need not fear comparison with any other institution.

In consequence of the usually crowded condition of Saint Yon, and the necessity of having additional sleeping accommodation, with greater space for employing the lunatics, a new asylum is now in course of construction, near the city, to contain 300 inmates and which will be appropriated exclusively to male patients; when the present institution will be restricted to insane females. According to report, the contemplated expense is calculated at 1,000,000 francs; but before the asylum is finished the outlay will doubtless, as often happens elsewhere, exceed the preliminary estimates.

In order to carry forward agricultural and horticultural labours, to which many psychological physicians in France justly attach great importance, a farm containing about ninety acres will be attached to the new establishment; and, judging from the plans kindly exhibited for my inspection, by the authorities, as also from the information communicated, the new asylum for the Lower Seine will be creditable to the department. Having the advantage of being specially projected to promote labour and employment amongst lunatics—so ably advocated by M. Parchappe—and being also superintended in its construction by the present Director of Saint Yon, Dr. De Boutteville, an able, although now a retired physician, and therefore fully cognizant of the requirements for such institutions, my present expectations respecting its beneficial results are somewhat sanguine. These anticipations I hope to see realized at some future period, when able to visit the new asylum, as also to thank again in person MM. De Boutteville, De Smyttère, and Mérielle, for the courtesies I experienced during my recent excursion.

### General Remarks.

When speaking of the medical staff of Saint Yon, I stated that four junior resident medical officers, called internes, are attached to the asylum, in which they are lodged, and have rations, besides receiving 400 francs per annum. Each interne may remain four years in office, but the average period varies from two to three years; and to be appointed, the candidate requires certain qualifications. During the time an interne continues at the asylum, he has excellent opportunities for obtaining knowledge in the best of all schools,—namely, from practical clinical experience—aided by the information also given by the physicians. Not only are the internes thus practically instructed to become future practitioners, but they in the mean time relieve the superior medical attendants of much labour and professional details. No asylum should, therefore, be without senior resident pupil officers; and having seen the inconvenience accruing from the want of similar officials, in some English as well as French institutions, I have more than ever become convinced of the great advantage of the above functionaries. In very few English asylums are there such appointments; nevertheless, the period is not distant when every asylum for lunatics in the British empire will have several professional attendants of this description, like the ordinary civil hospitals, which now possess dressers and house surgeons.

Feeling very strongly the importance and advantage of having, not only medical pupils, but also resident internes at every asylum for the insane, I must enlarge a little upon this interesting question, especially, as my early convictions have become materially strengthened, by resent observation and experience. From the facts recorded in previous pages, it will have been noticed as very remarkable, that wherever internes were officiating in any of the asylums described, restraint was less employed, and the patients there appeared even more tranquil; besides the great relief from the laborious details, which such an arrangement gives to the attending physician. The daily experience of those conversant with the insane, and especially of physicians habitually engaged in teaching, or lecturing to medical pupils in public lunatic institutions, fully bears out the opinion, that harm very seldom arises from the attendance of students, if properly regulated, the same as in ordinary hospitals. On this point, the views entertained by many practitioners of eminence might be quoted; but at present one reference will suffice, as the sentiments expressed are based upon long experience, both from treating insane patients, and in giving lectures on insanity.

The authority now referred to is M. Falret, the distinguished physician of La Salpêtrière, who observed, in a recent lecture he delivered, "that so far from being detrimental to insane patients, the attendance of pupils is often adjuvatory to their treatment; whilst clinical instruction might be as safely imparted, as in any ordinary hospital." M. Falret likewise made an important remark respecting French students, which would, I am sure, be equally applicable to Englishmen, under similar circumstances, viz. that in his opinion the pupils always conducted themselves in a satisfactory manner.

Timid and prejudiced objectors have said, no doubt, the practice of giving clinical instruction in a lunatic asylum would irritate and wound the feelings of insane patients; but this erroneous view is assumed rather from the observation of man in his sound, than in an abnormal mental condition. Hence the origin of much fallacious reasoning, by well-meaning and benevolent, but mistaken individuals. If completely insane, no injury whatever is thereby likely to supervene to the patient; although occasionally in certain cases, when approaching convalescence, the presence of pupils, in that particular dormitory, may sometimes do harm. Should this appear, discrimination then becomes necessary; of which the attending physician being the best judge, would, therefore, always act accordingly.

The attendance of pupils in the wards of a lunatic asylum, along with the usual medical attendant, cannot surely have any worse effect than in an ordinary hospital. Again, cases of interest would be often more minutely inquired into; their history, symptoms, and treatment investigated with greater care than, perhaps, they might be under other circumstances; from being thus exposed to the observation of intelligent students anxious to gain experience. For it is a common remark, that the sick poor are frequently better attended, and also more assiduously treated, when placed under the general observation of the profession, than otherwise; especially when a "chiel" is taking notes to print them afterwards. Instead of injury, benefit will often follow such a proceeding; and even should any inconvenience be occasionally the consequence of placing lunatic upon the same footing as general hospitals, that result cannot be at all compared in importance, with the many advantages which must then arise to the profession, the community, and still more, to every victim of insanity under treatment in public institutions.

Although unwilling to accumulate arguments upon a subject, which appears to all unprejudiced minds to be now settled satisfactorily, I must still crave the permission of readers to state, that an experienced professional friend, and resident medical officer of one of the largest asylums in England, and whose skill and judgment fully justify the high esteem in which he is held by all his acquaintance, candidly informed a county magistrate, also a friend of my own, who recently consulted him on the subject of an approaching medical election at another asylum, that when appointed to his present office "he previously never had the opportunity of feeling the pulse of a lunatic." It is unnecessary to say, that this gentleman is an earnest advocate for the system of instruction by lectures, respecting the nature and treatment of mental diseases; and has also materially contributed, by his individual exertions, to lessen that defect in medical education, which many other practitioners feel, and earnestly desire to see remedied.

When giving in previous pages the various statistical returns obtained from the eight public institutions mentioned in this communication, I considered it would be rather out of place to make special remarks upon the movement of patients in particular asylums, so as thus to avoid repetition; for these reasons, I purposely abstained from deducing any inferences, or enunciating general conclusions on such premises, until all the facts thus collected were placed in juxtaposition; believing that deductions, based upon an extensive series of facts, are more important, and in a higher degree conclusive, than if only drawn from a limited number of data; whilst they also give a more comprehensive outline of the chief phenomena. With this view, the following tabular statement has been compiled, in order to show the total number of patients admitted, cured, and died in each asylum during the year 1849, separately and collectively. The amount of inmates under treatment on the day I visited the various institutions is also stated; and as all the figures were obtained from official documents, they may be relied upon as accurate.

In regard, also, to a most important question,—namely, the actual number of patients reported under restraint, I can likewise say, upon that point, there is no exaggeration, as every figure contained in the subsequent table was written down at the time, from my own personal observation. This record of the amount of mechanical coercion, noticed in French insane establishments, doubtless will attract the special attention of British physicians, who generally entertain very different notions respecting the personal restraint of lunatics, from many of their brethren resident beyond the English channel; where great diversity of opinion prevails upon so vital a question, compared with that held in England. It should however be remembered, that padded rooms are very rarely met with in any French asylum; and also, that the camisole is the usual method of employing personal restraint, generally sanctioned by the medical officers; whilst it is sometimes applied as much for the safety of attendants, as of the patients; although some practitioners still think such treatment even beneficial in agitated cases. Intending again to allude to the important question of restraint, along with other subjects, I would now request the reader's attention to the following classified statement of facts and figures, which fully merit careful examination:—

TABLE *showing the movement of Patients in eight French Provincial Asylums, during* 1849. *Also the total Population of Lunatics, when visited in August or September of* 1850, *with the number of patients under restraint. Compiled by Dr. Webster.*

| Name of Asylum. | Movements of Patients in 1849. Admitted. | | | Cured. | | | Died. | | | Total Population in the Autumn of 1850. | | | Patients under restraint. | | |
|---|---|---|---|---|---|---|---|---|---|---|---|---|---|---|---|
| | M. | F. | Total. | M. | F. | Total. | M | F. | Total. | M. | F. | Total. | M. | F. | Total. |
| Bon Sauveur . | 60 | 63 | 123 | 42 | 30 | 72 | 31 | 21 | 52 | 302 | 390 | 692 | 6 | 12 | 18 |
| St. Meén . . | 47 | 44 | 91 | 22 | 15 | 37 | 24 | 25 | 49 | 143 | 168 | 311 | 3 | 2 | 5 |
| Nantes . . . | 70 | 56 | 126 | 25 | 25 | 50 | 56 | 44 | 100 | 181 | 210 | 391 | 9 | 12 | 21 |
| St. Gemmes . | 69 | 57 | 126 | 32 | 39 | 71 | 39 | 31 | 70 | 161 | 179 | 340 | 12 | 15 | 27 |
| Le Mans . . | 41 | 33 | 74 | 15 | 20 | 35 | 8 | 8 | 16 | 124 | 134 | 258 | 3 | 4 | 7 |
| Blois . . . | 26 | 60 | 86 | 13 | 10 | 23 | 8 | 7 | 15 | 46 | 78 | 124 | — | 3 | 3 |
| Orleans . . | 71 | 66 | 137 | 20 | 11 | 31 | 48 | 45 | 93 | 246 | 275 | 521 | 8 | 17 | 25 |
| St. Yon . . | 100 | 104 | 204 | 39 | 37 | 76 | 56 | 53 | 109 | 261 | 468 | 729 | 3 | 7 | 10 |
| Totals . | 484 | 483 | 967 | 208 | 187 | 395 | 270 | 234 | 504 | 1,464 | 1,902 | 3,366 | 44 | 72 | 116 |

According to the preceding summary, it appears, that the number of patients of both sexes received, during the year 1849, into the above eight provincial asylums for the insane, was almost identical; 484 lunatic males, and 483 females having been admitted. More men, however, were cured than women; 208 being of the former, to 187 of the latter sex; whilst the total deaths not only surpassed the aggregate recoveries, in both categories, but the proportion of fatal cases was greater amongst male than female patients; the difference being thirty-six fewer deaths amongst the latter division, although the gross amount of females under treatment was considerably more than the male inmates. These statements are interesting, and well deserve the attentive consideration of psychologists.

Indubitably, the great mortality recorded in the above table, was materially augmented by the recent epidemic cholera; hence, the results now reported, must not be held as true criteria of the usual annual average; still the ratio of deaths was large, speaking comparatively. Again, the total cures were almost forty-three per cent. amongst male patients, compared with the admissions; but only 38.71 per cent. amongst females, if similarly calculated. In reference to the amount of mortality, attention should be directed to that of Le Mans, as it furnishes a good index of the ordinary rate recorded in a provincial lunatic asylum, seeing no patient died of cholera at that institution; where, in an average population of at least 250 lunatics, the deaths, by all causes, amounted only to sixteen cases, during the entire year. Thereby making the ratio 6.20 per cent.; the number of persons discharged cured being, at the same time, nearly one-half the amount of admissions. Again, at St. Meen's asylum, if the twenty-one deaths reported by cholera be subtracted from the total forty-nine fatal cases stated in the previous table, the mortality, by all other causes, then becomes 9.31 per cent. amongst the residents. To pursue this limited mode of analysis any farther, at present, is unnecessary; whilst, to consider the total deaths now reported in the various asylums, as indicating ordinary occurrences, would be both unjust and erroneous. Foreigners might with equal justice assert, "London was a most unhealthy capital," because the mortality, of the same season, reached far beyond its usual amount; 27,109 persons having died in the metropolis of Great Britain, from all causes, during July, August, and September, of last year, instead of 13,503, which occurred in the parallel quarter of 1848; or of only 11,578, reported throughout the same districts, in the similar three months of the year 1850, just terminated.

Another feature in this table also deserves special remark,—namely, that female patients predominated, in respect of number, over the male lunatics in every asylum, especially in that at Bon Sauveur, and at Saint Yon; the excess, in the latter, being not less than 79.30 per cent. Even, if all the 3366 inmates are taken collectively, the difference, in the two sexes, is still nearly one-third; the numbers being 1464 male to 1902 female lunatics; thus giving 438 more patients of the latter than the former, and thereby indicating that now, in some districts of France, at least, insanity is a more frequent malady among women than men; the same, as I have heard, prevails throughout the whole country, as it is likewise in England; particularly amongst the metropolitan population. Various reasons are usually assigned for this decidedly greater liability of females than males to mental disease; but into that interesting question I will not enter farther, as it would be rather out of place to discuss such a subject at present; my chief object being to point out, rather than to explain, the above well-established tendency of mania.

Respecting the amount of mechanical restraint actually noticed in the different asylums, at the period of my visit during last August and September, the figures I have given in the table will, doubtless, attract much attention. Taken collectively, the numbers show, that forty-four male lunatics, out of 1464 then resident, were in camisole, some being also otherwise restrained; thereby giving one individual in restraint to every $33\frac{1}{4}$ male inmates; or three per hundred. Amongst the female lunatics, again, the proportion was somewhat larger; seventy-two persons of that sex, out of the total 1902 resident patients, being under mechanical coercion; thus making one female in restraint to every $26\frac{1}{3}$ inmates; or at the rate of 3.78 per hundred. In contrast with this report respecting the above-named French provincial asylums, I would now place an official statement of the practice pursued at Bethlem hospital, during the same period. At this establishment, where formerly the strait-waistcoat, with various kinds of personal coercion, were in even greater use, than on the other side of the English channel, *not one insane patient*, amongst an average population of 391 lunatics, was under constraint of any description, during the five weeks ending the 20th of last September; when I first revisited that institution, after my return from the continent, and which embraced the whole time referred to in this narrative.

Whilst describing some of the French lunatic institutions noticed in previous pages, allusion was made more than once to the deficient supply of water. This constitutes a serious evil in every public establishment, but especially in an asylum for lunatics, and contributes very materially to the production of sickness amongst the patients. Wherever a constant and abundant supply of good water exists, to carry away all refuse, and to promote personal cleanliness, as also for culinary or other purposes, then fevers and most zymotic diseases are much less likely to prevail, even when rife in the adjoining neighbourhood. Of course, other causes must not be overlooked, as they likewise exert great influence. Still, an abundant supply of water is essential; wherever large assemblages of human beings are congregated together, whether mentally sane or otherwise. This truth was remarkably illustrated at Bethlem hospital in the autumn of 1849; when cholera proved epidemic, and was very fatal in the immediate vicinity of that institution. In this large charity, not a single inmate was attacked by cholera, notwithstanding the hundreds, nay, even thousands, who became victims to that epidemic malady, within even a short distance of the building. When alluding to this remarkable exemption of Bethlem hospital from cholera, during the recent outbreak, and at the very time it proved so fatal in some French asylums for the insane, I stated in another publication, as an explanation of this phenomena, that here "there is a most abundant supply of pure water throughout the whole establishment, alike for baths, washing, and cooking. So copious, indeed, is the quantity, that in every water-closet, in consequence of a spring being attached to each door, whenever any person enters, the movement thus made simultaneously opens a valve; connected with a cistern, from which a torrent of water rushes into the seat; and this operation is repeated, when the patient retires. Further, through all the drains, water runs constantly; chloride of lime is also daily and freely used; and as every drain has been properly trapped, no odour ever prevails. A very deep artesian well—reaching to below the chalk—supplies the water required in the establishment; which a steam-engine pumps, at the rate of seventy gallons per minute, into the numerous reservoirs on the roof of the building, which are frequently cleansed, in order that the water may not be contaminated; hence, there is never any deficiency of this element, most essential in all institutions, but especially in asylums for the insane." The French physicians are fully aware of the vital importance of having an abundant supply of water in every public institution; and, to prove the existence of this feeling, I might again allude to the proposed erection of a steam-engine by Dr. Le Vincent, to pump water from the Loire, for the institution of St. Gemmes; as also to a similar proposal now in contemplation for the asylum at Blois, where a more copious supply is likewise much wanted.

Of late years, great progress has been made in France, respecting the nature, pathology, and medical treatment of mental diseases; whilst throughout Europe, few psychological physicians have done more in this important department of medical science, than several of the distinguished practitioners now attached to the lunatic establishments of the former country. To prove this assertion, it may be stated, that as all bodies are examined after death, in the public lunatic asylums of France, the opportunities for studying the pathology of mental diseases are consequently ample; of which the medical officers take every advantage, and also register the morbid appearances noticed on dissection.

In reference to another point, it ought, however, to be stated, although the physicians present regular reports of all proceedings in their respective asylums, to the higher official and administrative authorities, which contain very frequently much valuable information, with statistical details, and other data; these reports are, nevertheless, often lost to the profession, seeing they seldom appear in print, as in this country; but become buried, like many other public papers, in the archives of the Prefectures, the minister's bureau, or consigned to unmerited oblivion in some similar receptacle. Knowing the value of such useful documents in England, I think the Councils-General of departments in France should follow the system here adopted, as well as in America; knowing well, if the physicians of every French asylum for the insane published annually a detailed report of proceedings, the amount of highly useful and practical information thus supplied to the profession, would be very considerable. The instructive papers of this description, which MM. Parchappe, Bouchet, Aubanel, Billod, Morel, Giraud, Renaudin, and other experienced practitioners have given to the medical profession, conclusively indicate how much might be thus accomplished. The central government, with such excellent examples for their guidance, ought to sanction, if they did not absolutely require, for publication, a detailed official statement from every public and departmental asylum, throughout their entire jurisdiction.

Before taking leave of the medical officers of institutions for the insane in France, of whose zeal, ability, and devotion to the humane cause they have undertaken, it would be both easy and gratifying to speak at greater length; I must say unequivocally, the remuneration received is often too small, and by no means adequate to the labour performed, or the position they as physicians hold in society. Being in most cases debarred from engaging in private practice, the sole professional support these gentlemen have is their annual salary, and an official residence. Undoubtedly, in Paris, Rouen, and in some other cities, the physicians of public lunatic asylums are not always prevented from giving their medical services, and part of their time, to the public; which, of course often makes a great difference; still to pay some of the distinguished men I could name, but refrain out of delicacy to their feelings, with only 3000 or even 2400 francs per annum, is very illiberal, and by no means, sufficient remuneration; especially, where private paying patients are received into the asylum, of whom none could ever be attended before or afterwards, by the then resident physicians. Either the salaries now paid ought to be increased both at first, and afterwards, according to length of services; or private practice uniformly permitted, when not interfering with their official duties, which, of course, must always be duly performed. Wherever bad regulations prevail, they should be reformed; but if laws are good in Paris, the same become equally applicable to Nantes. Again, if private practice be permitted in Rouen, it cannot be wrong at Orleans. Further, Bon Sauveur with 692 insane patients, should have at least one resident physician, with four internes; quite as much as the neighbouring asylum of St. Yon, containing 729 inmates. Lastly, St. Gemmes Asylum, with 340 lunatics; and especially the Orleans institution, containing 521 insane patients, instead of only one resident medical officer, without, in both cases, even a single interne,—ought to have, if not two physicians, with two internes, certainly, three or four of the latter useful officers attached to each establishment.

In French asylums, there is I believe, no matron; or at least, no female attendant having the attributes of that important personage in English institutions; and which many consider an advantage in favour of our neighbours. A superior "sœur religieuse" possessing power over the other female attendants, is, however, not uncommon; whilst in the laundry, and other departments, there are various female officials; but the treatment and general management if the patients, whether on the male or female division, rests solely with the physician. In this respect, I consider the system pursued in the new French institutions well worthy of imitation, on the English side of the channel; where the matron sometimes becomes too important, from not being sufficiently under medical control, according to the opinion of many persons, well qualified to decide the point, from their own experience.

On the other hand, each departmental asylum has a director, who is a most important personage; being the chief superintendent of everything appertaining to the lay administration of the establishment. In some instances, the resident physician is likewise director; but in many asylums, the offices are entirely separate; which arrangement, to my mind, appears altogether the most eligible, as the attributes of both are incompatible, particularly in a large establishment. Under such circumstances, it must be allowed, that a physician whose mind is frequently engaged in financial questions, or if harassed by extensive commissariat duties, will not be able to find much leisure time for study and science. In an asylum, having a very limited number of inmates, the duties being in that case of a restricted description, the junction of the two offices may be permitted: and as such an arrangement will augment the remuneration of the physician, generally so underpaid, it has that advantage, without being then likely to prove injurious to the patients.

Believing it will be interesting to some English readers, to obtain a few general facts respecting the extent of accommodation at present available for the treatment of lunatics, belonging to the various departments of France; I would remark, that the institutions for insane patients, whether departmental, or those only forming a division of a general hospice, and lastly, those private establishments which now receive indigent patients, by arrangement with particular departments, recently amounted to 67; and were thus classed. The departmental asylums similar to Le Mans are 37; those forming only one division of a general hospice, as at Nantes, are eighteen in number; whilst twelve private establishments, like Bon Sauveur, now receive insane patients, by pecuniary agreement with neighbouring departments. Besides the above sixty-seven establishments for lunatics, there are also the three great public institutions of Paris; namely, Bicêtre, La Salpêtrière, and Charenton; thus making seventy asylums for the insane, throughout France, which are now placed more or less under the surveillance of government.

As might be expected, the total amount of lunatics in the above institutions is very large; the number being, according to the most accurate accounts I have been able to obtain, about 22,400; the majority of which are females. Besides the aggregate number now mentioned, the inmates of various private lunatic asylums throughout the country must be also taken into account, before we can correctly estimate the prevalence of insanity in France; which, although not so common a disease as in England, in regard to the population, is nevertheless very considerable; especially in the French metropolis. This is shown by the total admissions of insane patients during 1849, at Bicêtre, Charenton, and La Salpêtrière, which are reported to have been 1421 cases, taken collectively; the sexes being nearly equally divided. Again, in regard to recoveries, 474 patients were discharged cured from the above named three institutions, during that year, 235 being men, and 239 women; whilst the total deaths amounted to 949 individuals, comprising 334 male and 615 female lunatics. It should however be remembered, that this very large mortality amongst the insane women arose especially from cholera, which then prevailed at the Salpêtrière like a pestilence; 599 persons having died in this institution during 1849, by that epidemic.

Another feature in the movement of patients under treatment at these establishments should be likewise noticed; viz., the number of lunatics reported to have escaped from Bicêtre, Charenton, and La Salpêtrière during last year, which actually amounted to 35 individuals; 23 being men, and 12 women. In contrast with the above official statement of escapes effected from the three public lunatic asylums of Paris, it may be interesting to state, that from Bethlem hospital, during 30 years ending the 31st of last December, although 6952 curable insane patients had been admitted into the institution—consisting of 2781 men, and 4171 women, only twenty-three lunatics have escaped from the hospital, 15 being male, and eight female patients; most of whom, or twenty individuals, were however speedily brought back to the institution. It is also curious, if not instructive, to remark, that both in Paris, and at Bethlem, the number of male patients who effected their escape, was greater than the female; the proportion being two men to one woman in either instance.

Formerly, when restraint was much more frequently employed in the ordinary treatment of lunatics, than recently, escapes were then by no means uncommon. Indeed, the stricter insane patients were confined, and the oftener mechanical coercion was employed, escapes then became more numerous. For instance, during twenty years, ending the 31st December, 1769, when the strait-waistcoat, straw, and even chains were but too commonly the lot of unfortunate lunatics in this, as in nearly every asylum; it appears, amongst 3629 patients admitted into Bethlem hospital, fifty-five individuals escaped, forty-four being men, and only eleven women; thus giving one escape in every sixty-six admissions. More recently, and particularly when restraint of any kind is now very rarely employed, and the strait-waistcoat is unknown, escapes from the above institution have become of very rare occurrence. Thus, during thirty years, ending the 31st of December, 1849, although 6952 unable insane patients have been admitted, the whole of whom were recent cases, only twenty-two persons escaped, fifteen being male, and seven female lunatics; thereby making one evasion in every 316 admissions. If calculated in reference to sexes, the results then become very different; the escapes amongst male lunatics being actually one in every 185 of that sex admitted; whereas, it is only one in every 595 insane women; and if viewed comparatively, the proportion thus seems threefold greater, amongst male than female lunatics.

In my opinion, based upon these and other conclusive data, the more insane persons are restrained, the oftener attempts will be made to escape from confinement in any lunatic asylum; and I feel thoroughly convinced, were coercion less frequently employed, than unfortunately still prevails in some parts of Europe, escapes as well as suicides would not be so numerous. In reference to the fact of self-destruction being much more common amongst lunatics when mechanically restrained, than under the present very different system, I would mention, as a marked illustration of this great truth, that in Bethlem hospital, where personal coercion by various means was so generally employed,—namely, during the same twenty years already quoted, in reference to this subject, or prior to the 1st of January, 1770, at which period escapes were also common; eighteen patients actually committed suicide, consisting of six male and twelve female lunatics, which makes one case of self-destruction in every 202 admissions. Now, however, (and such a fact should be always remembered,) when the strait-waistcoat is unknown, and mechanical restraint of any description very rarely used, suicides, like escapes, have become much more infrequent. In proof of this opinion I may state, that during the last thirty years, notwithstanding mechanical coercion was more employed at the beginning of that period than towards its close, exactly eight suicides, of whom two were men and six were women, have been recorded amongst the whole 6952 curable insane patients under treatment in the hospital; being thus one case in every 869 admissions.

When noticing the peculiar feature exhibited at Bethlem, as also at the three Parisian public lunatic asylums, of male patients having much oftener effected their escape than females, it should be, on the other hand, remarked, that both recently, as also about the middle of last century, a greater number of women committed suicide at Bethlem Hospital than insane men; which propensity may be in part explained by the ascertained frequency of suicidal mania in the female oftener than in the male sex—the more acute feelings of the former, and their less self-command over actions which men seem in a higher degree able to control. Again, women being more domestic in their habits and conduct, feel less disposition to escape from their actual place of residence; whilst men, being comparatively more accustomed to change of place, to varied and active employments, and to be also much out of doors when in health, have become reconciled with greater difficulty to confinement in an asylum than women. However difficult it may be to explain such peculiarities in the two sexes, the facts now stated respecting the diversified disposition to escape and to suicide noticed in each sex appear, nevertheless, important; especially as they conclusively demonstrate, that the more frequently personal coercion of lunatics is employed, independent of many other evil consequences, so will casualties of the above description more likely supervene.

Notwithstanding it would prove both interesting and instructive to discuss the medical treatment usually pursued by physicians in the lunatic asylums of France, the length to which this paper has already extended prevents my now enlarging upon such an important topic, especially as some readers might consider it unnecessary. Nevertheless, I would briefly remark, that in addition to occupying and amusing the patients, great utility is attached in every institution to frequent baths, where the apparatus for bathing is always considerable—as at St. Yon, for instance, in which the bath-house is well worth examination. The douche does not appear to be so much employed as formerly—at least judging from my own previous observation; and it is only now used by way of punishment. The cold affusion seems, however, to be frequently prescribed in many of the French asylums. Hence, in refractory patients, who refuse to work when occupation is considered advisable by the physician, this form of bath proves frequently beneficial; and in many cases, where such cold affusion is employed, the patients are soon induced to commence the work they had previously refused to undertake; whereupon the medical attendant immediately stops any further affusion of cold water, as it is almost always found, that individuals so treated then keep their promise to resume employment. An instance of the kind, amongst others, may be here mentioned, since it shows the efficacy of a cold affusion-bath in refractory cases of insanity. One of the female inmates at St. Meen's Asylum having refused to continue some work, upon which she had been engaged the day previous, was ordered by her medical attendant to have the cold affusion. This remedy was accordingly applied in our presence, at the termination of the morning visit; as the order could only be executed, when the physician or an interne was actually present to watch the effect, and to stop any unnecessary application. Whilst the second bucket was being poured over this poor maniac's head—her body being then immersed in an ordinary bath, but so secured, that she could not escape the stream, or do any injury to herself, either with hands or limbs, she immediately called out, "*Grace, monsieur—je travaillerai.*" The further affusion of cold water was instantly interrupted, a few kind words were spoken to her by the interne by way of encouragement, and she was ordered back to the workroom. Subsequently, that gentleman remarked to me, that he confidently expected this patient would again return to her work as readily as three months before, when the cold affusion-bath was most successfully employed; since which, she had continued tranquil, and been diligent during the interval, until yesterday's outbreak. Other cases of a similar character might be quoted, but I refrain, believing they would be considered superfluous.

Influenced by the same motive, I do not intend to discuss the various remedies usually employed in cases of mania, farther than briefly to state, that the principles acted upon in France differ very little from those pursued by most English practitioners. Where physical disease prevails in any lunatic, of course the symptoms must be viewed much in the same light, as if the patient was mentally sane; with this essential caution, however, that great prudence must be always exercised in the adoption of debilitating treatment. Formerly, physicians, in their practice, were often disposed to consider mental diseases to exhibit an inflammatory diathesis. Hence bleeding, lowering the system, and depressing remedies, were much more frequently employed than at present. Nutritious food, warm clothing, cleanliness of person, with other prophylactic and hygienic measures, are, however, now generally considered of most essential importance; whilst it has been remarked, at the same time, that wherever the inmates of a lunatic asylum are under-fed, sickness and mortality will abound.

To prove that the sufficiency of food, and its good and nutritious quality, have much influence on the health of lunatics, I might mention a remarkable illustration which occurred at Bicêtre, during the first revolution, when the constituent assembly of France reduced the quantity of bread distributed to the inmates, from a kilogramme (two lbs. one-fifth oz.) to seven hectogrammes and a half (twenty-four ozs.), whereby a great number of the old convalescents relapsed into a state of raving madness. This system of retrenchment having been afterwards carried to a still lower grade, and even to half a kilogramme, the consequences to the poor maniacs were most disastrous; seeing that in two months of the fourth year of the Republic, twenty-nine deaths occurred among the patients; whilst, in the whole of the year *Two* (that is, when the allowance of bread was one kilogramme per patient), only twenty-seven deaths were reported during the entire twelve months. These facts furnish a very instructive lesson regarding the dietary of insane patients, and they point out the necessity of attending to the kind and quantity of nutriment, which the inmates of lunatic asylums ought to receive. For whatever may be the moral or remedial treatment pursued, unless due attention is paid to such an important point as the food and regimen of insane patients, any plan of management, however beneficial it might otherwise prove, will not be likely to realize the expectations of relatives, or to fulfil the wishes of a conscientious practitioner.

To sum up succinctly the principles a practitioner ought to follow in the management of lunatics, I would say insane patients must be treated as much as possible like rational beings, consistent with their own safety or that of others; and as the mind of such persons resembles, in some degree, that of a child, kindness, with decision, regulated by justice, ought to influence the actions of every attendant. No promise should ever be made to a lunatic which is not strictly kept; the feeble intellect of the afflicted sufferer must be always treated, as if it were a tender plant, and so kept as much as possible devoid of every external source of excitement. Subsequently, and wherever practicable, the patient should be employed in bodily labour; or, at least, an attempt made to engage the remaining faculties in some amusement or intellectual occupation, in order thereby to draw the maniac's debilitated mind from its usual morbid channels, but especially, from those notions and objects which have contributed to produce mental disease. Experience invariably teaches, wherever the physician is able to divert the patient's ideas—however feeble these may appear—from previous morbid cogitations and reminiscences, so as to fix attention upon new subjects of thought or occupation, any plan of treatment, whether moral or medical, then considered appropriate, will more likely prove efficacious, and so promote future convalescence.

Although the laws now in force respecting the administration of French lunatic asylums have conferred great benefits upon the country, and much improved the condition of the inmates, especially in the departmental institutions for the insane recently constructed, still, like our own lunacy enactments, they are capable of improvement. Into these important questions it would be rather out of place now to enter at any length; nevertheless I cannot avoid observing, that here, as in most public departments of France, the central government, whilst it exercises general control over the provincial authorities, and very properly so, in many cases has too much power in reference to details, and in the appointment of the superior officials. Again, the local administrations sometimes interfere with the functions of the medical officers, more than is judicious; whilst these have not always sufficient authority over the non-medical treatment of the patients, and over their male or female attendants. Further, the numerous and varied duties required from the physicians are often most laborious, and frequently seem like the work of a clerk in a banking-house or public office, rather than that of a man of science, whose time and mental energies should be almost exclusively dedicated to the cure of disease and his medical studies, instead of to manual labour.

As the minister of the interior nominates the physicians to all public asylums, it thence happens, that occasionally the local authorities are inimical to the nominee of the minister, especially if he be a stranger to the department. It likewise sometimes occurs, that the members of the governing body of a provincial institution wish a friend of their own to procure the appointment, whereby jealousy, and even dislike, is felt towards the stranger thrust upon an unwilling council of administration. I might give instances to illustrate such consequences, but refrain, as personalities are objectionable. Nevertheless, one well-known case might be now mentioned, wherein the minister exercised his power in reference to a very distinguished individual, which shows the way the system may be made to work—I allude to the treatment experienced by my excellent and learned friend M. Foville. This well-known physician and eminent physiologist, formerly physician to Saint Yon Asylum, and latterly of Charenton, was summarily deprived of that valuable and important appointment, about two years and a half ago, by an ephemeral minister of the day, who had clutched the seals of office for a time, and then used his temporary power against a long-tried public servant, that happened to be a friend of, and hence patronized by, the Prince de Joinville.

In a provincial asylum I could name, another minister of the interior having appointed a resident physician—a man of experience, but still a perfect stranger to the department, instead of a local candidate, the new official, so far from meeting with cordial support in the performance of his duties, was long looked upon with coolness, and considered an intruder; whilst his professional brethren in the locality never would associate with the favoured protegé sent from head-quarters. At Bon Sauveur, on the other hand, neither resident physicians nor internes have been appointed, notwithstanding the size of the asylum, and although the law of 1838 expressly says, there ought to be at least one resident medical officer. Again, neither at Orleans nor St. Gemmes are there any internes, who prove, everywhere, very useful officials. Why these discrepancies exist it is difficult to explain, nor is it within my province to say more, than that such anomalies require amendment.

Occasionally, however, interference with the medical officers may be carried too far, whereby the welfare of the inmates is apt to suffer, of which the following examples may be given as illustrations. Some time ago the medical attendant of a provincial asylum, who took much interest in the dietary of his patients, when going round the wards, and after tasting the soup and rice then served to a patient, one day said, in the hearing of the attendants, that "the former was too salt, and the latter not sufficiently boiled." This remark being reported to a local dignitary, it gave great offence, as he thought the physician was going out of his province, and accordingly brought the affair before the council of administration. This lèd to an investigation and correspondence, which was ultimately referred to the central government at Paris, to settle the disputed jurisdiction. The other case of interference happened in my own presence, and therefore I am able to vouch for its accuracy. When perambulating one of the provincial asylums, along with the responsible medical officer, on entering a court-yard containing some excited female lunatics, we were accosted by the nurse in charge of that division in tears, who said, that "she had been dismissed, and was then going tò leave." The nature of her fault, or who had turned her off, were alike unknown to the physician, or whether any successor had been in the meantime appointed to attend the patients. There arose plenty of talk in the ward, which we ultimately left for another part of the building. Very soon afterwards, having occasion to repass through the same court-yard, I was surprised to see one of the patients removing the camisole from another inmate, and that all the residents were now left to themselves, without any attendant; as the party dismissed had actually gone away the moment our backs were turned. So far from thinking the authority of any resident or attending responsible medical officer should be restricted, in whatever appertains to the management and treatment of the inmates of a lunatic asylum, I consider his power should be almost unlimited; the financial and material administrative arrangements being, of course, in other hands. In most of the newly-constructed departmental institutions the system, in this respect, is excellent, and, as I have already said, well worthy of imitation by other establishments.

Before concluding the present narrative—now submitted to the readers of Dr. Winslow's *Psychological Journal*—I should neither do justice to the many excellent professional and official persons with whom I had the good fortune to come in contact, during my recent tour, nor even to my own individual feelings, were the civilities and kindness which I uniformly experienced passed over in silence, and thus appear as if they were already forgotten. On the contrary, when now taking a retrospective glance of my proceedings, and remembering the trouble often given to physicians, officers, and others, by my numerous inquiries, I cannot sufficiently thank all the gentlemen, who afforded me every facility to obtain information. To each, individually, I would again tender my best acknowledgments for the many courtesies I received; but especially to MM. Levincent and Chambeyron, of whom I shall always retain the most agreeable recollections. To my distinguished friend M. Parchappe—one of the inspectors-general of lunatic establishments in France—I am even more particularly under obligations, as it was through his instrumentality, by kindly furnishing me with a general letter of introduction to the physicians and directors of every asylum I might choose to visit, that the advantages alluded to above were so readily procured. Should any of the criticisms made in reference to some of the institutions described in my Notes seem somewhat severe, the blame does not, in justice, rest with the medical officers mentioned; on the contrary, by these gentlemen many of the great improvements recently accomplished were proposed, besides those still in progress. Nay, I would again repeat, were every establishment more under the immediate control of the responsible medical attendant—were these officers more numerous, *better paid*, and all had internes, then truly some of the remarks contained in previous pages would never have been uttered; or, if expressed, could have rested upon no foundation.

---

The patience of most readers of these desultory Notes of my recent visit to several provincial asylums in France being, perhaps, already exhausted, I will now bring the present rather lengthened communication to a close, by digressing from professional questions, to notice one or two subjects of general interest to most travellers. The first relates to passports—that necessary, yet very great nuisance to every Englishman going abroad. And although much has been recently said upon this matter, in one respect the system is even worse than previously, as I can state from personal experience. Thus, during all the former journeys I have made to France—and they are numerous—whether under the elder Bourbons, the Citizen King, or even the new Republic, until the present season my passports were always delivered *gratis* by the French authorities in London. This year, however, before that still indispensable document could be obtained, *five shillings* were demanded, and paid to the official, who said, "Such was the law and custom." That, no doubt, might be so; but the alteration indicated neither liberality nor reform, about which, of late, there has been frequently much conversation. In financial respects, certainly, the change is advantageous; and as the number of my recent passport was 3,227, thus upwards of 830*l.* had been already received for permissions to travel in France, thereby making a considerable item for papers which were formerly given gratuitously. This tax, with other causes, have, however, materially diminished the crowd of English tourists; as shown by the fact, that in the same season, and exactly at four days' earlier date, in 1847, during Louis Philippe's reign, when I also visited Paris, the figures written on my French ambassadorial passport are 7098; or actually more than double the issues during the current year. These apparently unimportant statistical data, however, merit notice; and although neither medical nor scientific, I have been induced now to mention them, as apt illustrations of recent political changes, and their consequences.

But to prove still further, that English travellers are much less frequently met with in some parts of France than formerly, another circumstance may be also stated, which appeared at the time, as now, rather remarkable, and constitutes important additional evidence in support of such an opinion; whilst it also seems an appropriate concluding paragraph to the present report of my recent excursion. The occurrence referred to is now related more willingly, seeing the observation differs from that often made by persons visiting France, at the usual touring season, and may be thus described:—From the period of leaving the sea coast, and during the whole of my wanderings in Normandy, Brittany, on the banks of the Loire, and through several neighbouring departments, which extended to nearly eight hundred miles, and occupied some time, *I never met a countryman, spoke a word of English, or saw any British newspaper*, notwithstanding the whole journey was made by railway, in steam-boats, or the ordinary conveyances; whilst I often frequented cafés, tables-d'hôte, with other usual places of public resort, and was always on the move, or in society. Besides these opportunities of meeting strangers, I also visited the crowded exchange of Nantes, at the hour when merchants, besides men of many nations, do congregate, and likewise passed a day at Blois, formerly a favourite residence for self-expatriated and renegade Britons, whose object often was, rather to enjoy cheap luxurious living, than to advance the interests of their native land; although from thence, in the aggregate, they derived a large revenue, to be thus expended in a foreign country. Notwithstanding all this, until my arrival in Paris, subsequent to the visit I have recorded in these pages, the above rather singular, but, on my part, involuntary, proceedings continued without interruption. However, when returning from Nôtre Dame Cathedral, having met an eminent London physician, a friend and near neighbour, who had, like myself, come to pass his holidays on the soil of "La Belle France," the spell which hitherto, figuratively speaking, seemed to hover round all my varied movements, and even to influence external events, was at last broken, whereby I now resumed my native language, and with that event again became an Englishman.

---

